# Identification and Determination of Impurities in a New Therapeutic Agent for Fatty Liver Disease

**DOI:** 10.1155/2023/3116223

**Published:** 2023-08-04

**Authors:** Huihui Shao, Jing Feng, Hanyilan Zhang, Yuanyuan Zhang, Tong Qin, Yuhua Hu, Wenxuan Zhang, Tiesong Wang, Song Wu, Qingyun Yang

**Affiliations:** ^1^State Key Laboratory of Bioactive Substance and Function of Natural Medicines, Institute of Materia Medica, Chinese Academy of Medical Sciences and Peking Union Medical College, Beijing 100050, China; ^2^NMPA Key Laboratory for Research and Evaluation of Generic Drugs, Beijing Institute for Drug Control, Beijing 102206, China

## Abstract

Methyl 7,7′-dimethoxy-5′-(morpholinomethyl)-[4,4′-bibenzo[d][1,3] dioxole]-5-carboxylate methanesulfonate (IMM) is an innovative drug for the treatment of nonalcoholic fatty liver disease (NAFLD) owing to its high efficacy and low toxicity. In this study, five minor impurities (I, II, III, IV, and V) were identified and analyzed using spectroscopic evidence, chemical synthetic methods, and liquid chromatography-tandem mass spectrometry (LC-MS/MS). The impurities included hydrolysates and oxidation by-products extracted from both the drug in its final formulation and during synthesis. Toxicity prediction revealed potential carcinogenicity of impurity V containing an N-oxygen fragment. A reliable and selective HPLC method for the quantitative analysis of impurities I–IV and a sensitive HPLC-MS/MS method for potential genotoxic impurity V were developed and optimized. The methods were validated based on the International Council for Harmonization guidelines. Satisfactory linearity was obtained for the analytes over the range of 0.1–2.0 *μ*g/mL for impurities I–IV and 0.3–30.0 ng/mL for impurity V, and in all cases, the fitting correlation coefficients exceeded 0.999. The obtained limits of detection values were 0.05 ng/mL and 0.005 *μ*g/mL for impurity V and impurities I–IV, respectively. The precision and repeatability of the methods were less than 1.08% and 8.72% for each impurity. The recovery percentages of all impurities were in the range of 91.18%–111.27%, with the relative standard deviation of less than 3.69%. The greenness assessment of the HPLC method and the HPLC-MS/MS method were evaluated by using AGREE software with a score value of 0.72 and 0.68, respectively. The recommended procedures that were accurate, specific, and ecofriendly were applied to the existing active pharmaceutical ingredients of IMM, and they generated satisfactory results.

## 1. Introduction

Nonalcoholic fatty liver disease (NAFLD), discovered in 1980 [1, 2], is a prevalent chronic liver disease characterized by the accumulation of fat in the liver. The prevalence of NAFLD in Asia was 29.62% between 1999 and 2019, and epidemiologic data revealed a sharp rise in the prevalence of NAFLD due to rapid urbanization and the global obesity epidemic [3, 4]. As of yet, no drugs for the treatment of NAFLD have been approved for Phase II and Phase III clinical trials by the U.S. Food and Drug Administration, the European Medicines Agency, and the National Medical Products Administration [5]. Bicyclol is a collateral phenyl-structured drug widely applied for the treatment of chronic hepatitis, which has been approved in China since 2004 [6]. However, due to the poor solubility and bioavailability of bicyclol, the structural optimization of bicyclol was carried out in our group's previous research. Notably, methyl 7,7′-dimethoxy-5′-(morpholinomethyl)-[4,4′-bibenzo[d][1, 3] dioxole]-5-carboxylate methanesulfonate, or IMM for short, was synthetized successfully by our research group [7, 8] (its spectral data are shown in Supplementary Materials S1). Preclinical studies have suggested that IMM, the bicyclic morpholine mesylate of bicyclol, has better pharmacological activities and pharmacokinetic properties than bicyclol in the aspects of liver protection and therefore holds great promise as a useful drug for treating NAFLD in the future. Moreover, as a methanesulfonate drug, the residues of methyl methanesulfonate and ethyl methylsulfonate were well below control limits [9]. IMM is currently in Phase I clinical trials.

**Figure 1:**
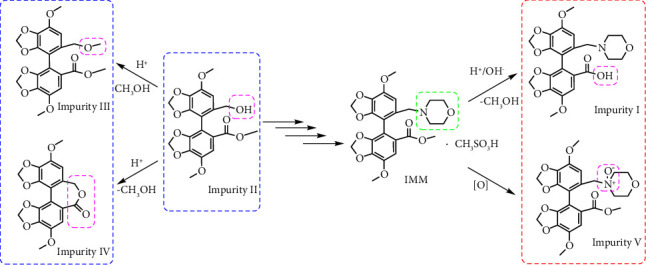
Synthetic process of impurities I–V.

In a preliminary study, we detected four impurities (I–IV) during the synthesis of our IMM active pharmaceutical ingredient (API). In addition, under the strong oxidation conditions of the forced degradation test, an unknown impurity exceeding the identification limit was found and named impurity V. Through high-performance liquid chromatography-ultraviolet spectrum (HPLC-UV) and liquid chromatography-tandem mass spectrometry (HPLC-MS/MS), we further analyzed each impurity's contents and possible structures. The chemical structures of impurities I, III, IV, and V were identified through synthesis and isolation. The possible pathways of impurity formation are shown in Figure 1.

The critical quality attributes proposed in quality by design are measures of drug quality [10–16], especially the investigation of related substances [17, 18]. The presence of impurities, even in small quantities, can influence the safety and efficacy of drugs. The most crucial factors of pharmaceutical analysis are the stability and impurity profiles of novel therapeutic compounds [19], and techniques that comply with green analysis must also always be taken into consideration and employed [20]. Thus, it is necessary to develop sensitive, accurate, valid, and economical methods for the reliable estimation of impurities in IMM. Among the five impurities mentioned previously, impurity V is nitrogen oxide with strong electrophilicity. Therefore, impurity V was controlled below the threshold of toxicological concern (TTC) level for safe administration of the drug clinically, cited at 1.5 *μ*g/day for long-term treatment [21]. In consideration of the maximum daily dosage (50 mg), the estimated permitted level of impurity V in the drug IMM is set to 30 ppm.

In accordance with the limit requirements of these impurities, an accurate, specific, convenient HPLC method was established to determine impurities I–IV, and a highly sensitive and reliable HPLC-MS/MS method was developed successfully for the analysis of impurity V at a 30 ppm level due to the low sensitivity of the UV detector. The proposed methods were validated in accordance with ICH guidelines [22–26], and the greenness assessment of two methods was evaluated by using AGREE software [27, 28]. The proposed HPLC method and the HPLC-MS/MS method represent favorable protocols in quality control and in-process monitoring during pharmaceutical manufacturing. The findings could provide reliable technical reference and scientific basis for the safe long-term clinical application of this product.

## 2. Materials and Methods

### 2.1. Materials, Chemicals, and Reagents

IMM substances (batch nos. 20211001, 20211101, and 20220622) and impurity I and impurities III–V were produced in the author's laboratory. Impurity II (purity 99.64%) was provided by Beijing Union Pharmaceutical Factory (Beijing, China). Acetonitrile (ACN), triethylamine (TEA), and LC–MS grade methanol (MeOH) were purchased from innoChem Science and Technology Co., Ltd. (Beijing, China). LC–MS grade formic acid (FA) was procured from Honeywell (Muskegon, MI, USA). Ammonium formate and hydrogen peroxide (H_2_O_2_) were purchased from Sinopharm Chemical Reagent Co., Ltd. (Beijing, China). Ultrapure water was acquired from a Milli-Q water purification system (Millipore, Bedford, MA, USA). The distilled water used was Wahaha purified water.

### 2.2. Conditions for the Determination of Impurities I–IV (HPLC Method)

HPLC analysis was performed on a Thermo Ultimate U3000 liquid chromatograph equipped with a DAD detector (Thermo Fisher Scientific Co., Ltd.). The liquid chromatography separation of IMM and impurities I–IV was performed using an ODS C_18_ column (4.6 mm × 250 mm, particle size of 5 *μ*m) maintained at 35°C. Acetonitrile (ACN) was selected as mobile phase A, and mobile phase B was a mixture of 0.1% (v/v) formic acid (pH adjusted to 4.0 with triethylamine) and ACN in a constant proportion of 70 : 30 (v/v). The following gradient was used for a 40 min sequence: the percentage of mobile phase B was held at 100% for 15 min, decreased from 100% to 70% between 15 and 30 min, and then held at 70% between 30 and 40 min. The flow rate was kept at 0.7 mL/min, the injection volume was 20 *μ*L, and ultraviolet (UV) detection was carried out at 230 nm.

The mixed solvent was acetonitrile, and water was mixed at a ratio of 70 : 30 (v/v). A sample solution of IMM was prepared at 0.5 mg/mL in a mixed solvent. Standard mixture solutions of impurities I–IV were prepared at 0.5 *μ*g/mL. The concentrations of the standard mixture and sample solutions were optimized to achieve the desired signal-to-noise ratio (S/N) and optimal peak shape. All samples and standard solutions were filtered through 0.45 *μ*m membrane filters before analysis.

### 2.3. Conditions for the Determination of Impurity V (HPLC-MS/MS Method)

Determination of impurity V was performed using an Agilent 1290 Infinity II HPLC system and a 6495 triple-quadrupole tandem mass spectrometer (Agilent Technologies, Inc., Santa Clara, CA, USA) equipped with an electrospray ionization (ESI) device. An Inertsil ODS-3 column (3 mm × 150 mm, particle size of 3 *μ*m) was operated at 35°C for separation. The mobile phase was a mixture of 2.5 mmol/L ammonium formate aqueous solution, containing 0.05% formic acid (mobile phase A) and methanol (mobile phase B) in a constant proportion of 72 : 28 (v/v) at a flow rate of 0.4 mL/min. The injection volume was set to 5 *μ*L. The parameters for positive ESI analysis were as follows: drying gas flow of 11.0 L/min, nebulizer pressure of 25 psi, gas temperature of 150°C, and spray voltage of 3000 V. Quantification was conducted using multiple reaction monitoring modes (MRMs). The MS/MS transition and capillary voltage (CE) conditions were as follows: 476.1 ⟶ 373.2 (*m*/*z*) (CE: 10 eV) for impurity V and 460.1 ⟶ 341 (*m*/*z*) (CE: 20 eV) for IMM.

Stock standards of impurity V were prepared at a concentration of 50 *μ*g/mL in methanol. Subsequently, a standard solution was obtained at 3 ng/mL by diluting the stock standards with 30% methanol aqueous solution. The sample solution of IMM was freshly prepared at 0.1 mg/mL. First, approximately 20 mg of IMM was accurately weighed, transferred to a 100 mL volumetric flask, and added to 75 mL of 30% methanol aqueous solution. Next, the solution was vortexed for 1 h until the sample was completely dissolved and then diluted to 100 mL with 30% methanol aqueous solution.

### 2.4. Validation of the HPLC Method

The validation of the proposed method for quantification of impurities I–V was performed based on the criteria of ICH Q2 and ICH Q3A, including specificity, limit of detection (LOD), limit of quantification (LOQ), linearity, accuracy, precision, and stability.

#### 2.4.1. Specificity

The specificity of the proposed HPLC method was assessed using forced degradation studies on the sample solution (0.5 mg/mL) by evaluating whether the blank solutions and other degraded impurities affected the main peak and known impurities. In the peak purity test, a diode array detector (DAD) was adopted and peak homogeneity was analyzed. Forced degradation studies were performed under the following five conditions:

1. *Acid Hydrolysis.* Ten milligrams of IMM was weighed, transferred to a 20 mL volumetric flask, added to 10 mL of the mixed solvent and 2.0 mL of 1.0 M HCl, and stored at 60°C for 24 h. Subsequently, the solution was neutralized with 1.0 M NaOH and then diluted to 20 mL with the mixed solvent.
2. *Basic Hydrolysis.* Ten milligrams of IMM was weighed, transferred to a 20 mL volumetric flask, added to 10 mL of the mixed solvent and 2.0 mL of 1.0 M NaOH, and stored at 60°C for 24 h. Subsequently, the solution was neutralized with 1.0 M HCl and then diluted to 20 mL with the mixed solvent.
3. *Oxidative Degradation.* Ten milligrams of IMM was accurately weighed and transferred to a 20 mL volumetric flask, then added to 10 mL of the mixed solvent and 1.0 mL of 30% H_2_O_2_, and stored at 60°C for 24 h. Subsequently, the solution was diluted to 20 mL with the mixed solvent.
4. *Thermal Degradation.* Ten milligrams of IMM was transferred to a 20 mL volumetric flask and exposed at 60°C in an oven for 30 days. Subsequently, 10 mL of the mixed solvent was added, and the solution was diluted to 20 mL to achieve a concentration of 0.5 mg/mL of IMM.
5. *Photodegradation.* Ten milligrams of IMM was transferred to a 20 mL volumetric flask and exposed to light of 4500 Lx ± 500 Lx for 30 days. The sample was horizontally positioned to provide the maximum area of exposure to the light source.

#### 2.4.2. System Suitability

System suitability was evaluated by injecting the mixture solution containing IMM (0.5 mg/mL) and impurity I (0.5 *μ*g/mL). The resolution between peaks of IMM and impurity I should not be less than 3.

#### 2.4.3. Linearity

A calibration plot was prepared with seven concentrations containing impurities I–IV in the range of 0.1–2.0 *μ*g/mL to establish linearity. The peak areas of the chromatograms were plotted against the respective concentrations of impurities to obtain the curves, and calculation of the regression line was performed using the method of least squares. The intercept, slope, and correlation coefficients were calculated using linear regression. A minimum of three measurements were performed per sample.

#### 2.4.4. LOD and LOQ

The LOQs and LODs of impurities I–IV were assessed by determining the S/N ratios of 10 : 1 and 3 : 1, respectively. The values were investigated using the minimum solution concentration (0.1 *μ*g/mL) under linearity, and the solutions were diluted with the mixed solvent stepwise. The diluted solutions were separately injected into the chromatograph.

#### 2.4.5. Accuracy

The accuracy of the HPLC method was evaluated through spiked recovery experiments by using three concentration levels, and the results were expressed as the percentage of the mean recovery and relative standard deviation (RSD%) for each concentration level. Sample IMM solutions (0.5 mg/mL) at the test concentration containing four impurities at low (50%), medium (100%), and high (150%) levels were prepared and analyzed (*n* = 3, per level). The recovery was calculated by the percentage of actual concentration and theoretical concentration by using an external standard method.

#### 2.4.6. Precision and Stability

Precision was determined using standard mixture solutions (*n* = 6) containing four impurities at a concentration of 0.5 *μ*g/mL. Repeatability was evaluated by the sample solution of IMM (0.5 mg/mL) added with known concentrations of the standard mixture solutions (0.5 *μ*g/mL), and six solutions were prepared in parallel to obtain repeatability. Solution stability was established by analyzing the solution at different time intervals (0, 2, 4, 8, 12, and 24 h) at room temperature. The results were summarized by calculating the RSD% value.

### 2.5. Validation of the HPLC-MS/MS Method

The proposed HPLC-MS/MS method was validated in terms of specificity, linearity, precision, accuracy, and stability. Specificity was determined by injecting the blank, mixed solution of IMM and impurity V (3 ng/mL), and the solution containing individual impurity V at a concentration of 3 ng/mL and IMM at a concentration of 0.1 mg/mL. The mixed solution of IMM and impurity V (3 ng/mL) was also used as a system suitability solution. A calibration plot was prepared by analyzing seven standard solutions of impurity V in the concentration range of 0.3–24.0 ng/mL to establish linearity. Then, 0.3 ng/mL of the solution was diluted stepwise with 30% methanol aqueous solution, and LOQ and LOD were defined as the concentrations that could be detected and could yield S/N ratios of 10 : 1 and 3 : 1, respectively. The verification procedures for precision and repeatability were consistent with those for the HPLC method. Precision was determined using the standard solution of impurity V at a concentration of 3 ng/mL (*n* = 6). Repeatability of this method was determined with sample solution (0.1 mg/mL) of IMM, and six solutions were prepared in parallel for this purpose. The stability of the solution under repeatability was observed at different time points under room temperature. The results were summarized by calculating the RSD% value. The accuracy of the method was determined by performing spiked recovery experiments using three concentration levels. Sample IMM solutions (0.1 mg/mL) at the test concentration containing impurity V at low (50%), medium (100%), and high (150%) levels were prepared and analyzed (*n* = 3, per level). The recoveries were calculated by comparing the actual and theoretical concentrations.

### 2.6. Isolation and Synthesis of Impurities

#### 2.6.1. Synthesis of 7,7′-Dimethoxy-5′-(Morpholinomethyl)-[4,4′-Bibenzo[d][1,3]Dioxole]-5-Carboxylic Acid (Impurity I)

A mixture of IMM (300 mg, 0.654 mmol) and NaOH (261 mg, 6.54 mmol) in tetrahydrofuran (10 mL) was added to 1 mL of water. The reaction mixture was stirred at room temperature for 8 h, followed by the addition of hydrochloric acid (1 M) for a final pH of 7.0. Then, the reaction solution was filtered, and the filter cake was washed with water to obtain impurity I as a white solid at 64% yield. HPLC-purity is 96.78%. ^1^H NMR, ^13^C NMR, HR-ESI-MS spectral data, and chromatogram of purity analysis are shown in Supplementary Materials S2 and S3.

#### 2.6.2. Synthesis of Methyl 7,7′-Dimethoxy-5′-(Methoxymethyl)-[4,4′-Bibenzo[d][1,3]Dioxole]-5-Carboxylate (Impurity III)

Impurity II (400 mg, 1.0 mmol) in methanol (10 mL) was stirred, and methanesulfonic acid solution (0.1 mL) was added. The reaction mixture was stirred and refluxed for 2 h to react completely. After cooling, the reaction mixture was concentrated in vacuo to give a residue, which was dissolved in dichloromethane (20 mL) and washed with saturated NaHCO_3_ solution. The combined organic phase was dried over anhydrous Na_2_SO_4_, filtered, and evaporated under vacuo to obtain impurity III as a white solid at 75% yield. HPLC-purity is 99.18%. ^1^H NMR, ^13^C NMR, HR-ESI-MS spectral data, and chromatogram of purity analysis are shown in Supplementary Materials S6 and S7.

#### 2.6.3. Synthesis of 4,10-Dimethoxy-[1,3]Dioxolo[4′,5′:3,4]Benzo[1,2- c][1,3]Dioxolo [4′,5′:5,6] Benzo[1,2-e]Oxepin-6(8H)-One (Impurity IV)

Impurity II (400 mg, 1.0 mmol) in tetrahydrofuran (10 mL) was stirred, and HCl solution (0.1 mL, 1 M) was added. The mixture was stirred at room temperature for 2 h. After that, the reaction mixture was concentrated in vacuo to give a residue, which was dissolved in dichloromethane (20 mL) and washed with saturated NaHCO_3_ solution. The combined organic phase was dried over anhydrous Na_2_SO_4_, filtered, and evaporated under vacuo to produce impurity IV as a white solid at 75% yield. HPLC-purity is 99.66%. ^1^H NMR, ^13^C NMR, HR-ESI-MS spectral data, and chromatogram of purity analysis are shown in Supplementary Materials S8 and S9.

#### 2.6.4. Isolation of 4-((7,7′-Dimethoxy-5′-(Methoxycarbonyl)-[4,4′-Bibenzo[d][1,3]Dioxol]-5-yl)Methyl)Morpholine 4-Oxide (Impurity V)

IMM (5 g, 9.0 mmol) was dissolved in 30% H_2_O_2_ (25 mL) and stored at 60°C for 20 days. Afterward, dichloromethane (60 mL) and water (25 mL) were added and extracted twice, and the organic layer was washed with saturated NaCl and dried over anhydrous Na_2_SO_4_. Then, the combined organic phase was subjected to silica gel (300–400 mesh, 20 g) column chromatography with dichloromethane/methanol (30 : 1) as the eluent to produce a white solid (100 mg). The solid was further purified by preparative thin-layer chromatography with dichloromethane/methanol (20 : 1) as the eluent to obtain impurity V at 1.4% yield. HPLC-purity is 95.63%. ^1^H NMR, ^13^C NMR, HR-ESI-MS spectral data, and chromatogram of purity analysis are shown in Supplementary Materials S10 and S11.

### 2.7. Computational Toxicology of Impurity V

Online ADMETlab 2.0 [29], preADMET [30, 31], and pKCSM [32] computer programs were used for toxicity prediction for the AMES test and evaluation of the potential tumorigenic effect of impurity V. The chemical structure was described with SMILES language. In addition, Toxtree software was employed to predict toxicity with structurally alerting functional groups.

### 2.8. Greenness Evaluation of the Proposed Methods

AGREE software was used for evaluating greenness of the proposed HPLC method as well as the HPLC-MS/MS method.

## 3. Results and Discussion

### 3.1. Detection and Identification of Impurities

**Figure 2:**
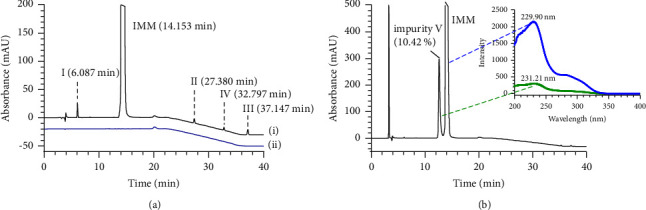
HPLC chromatogram of IMM and its impurities: (a) (i) IMM crude substances (0.5 mg/mL) and (ii) blank; (b) oxidative degradation of IMM (30% H_2_O_2_).

As shown in Figure 2(a)(i), four inconspicuous impurity peaks were observed at retention times (Rt) of 6.087 (I), 27.380 (II), 37.147 (III), and 32.797 min (IV), except for IMM (Rt = 14.153 min), during the HPLC analysis of IMM crude substances. To clarify these impurities, we proposed and synthesized several possible by-products expected during synthesis reaction of IMM. The Rt values of the four impurities in HPLC were compared with those of the reactants in the preparation of IMM. The comparison analysis revealed that impurity I was the hydrolysis product of IMM, impurity II was from the starting material (bicyclol), impurity III (bicyclol methyl ether) was formed by bicyclol and methanol under acidic conditions, and impurity IV (bicyclol lactone) was a by-product formed by removing methanol from the intramolecular part of bicyclol.

**Figure 3:**
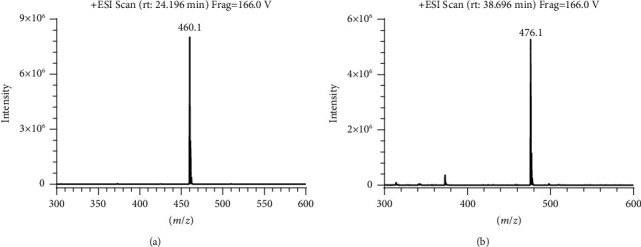
HPLC-ESI-MS spectra of (a) IMM (*m*/*z* 460.1) and (b) impurity V (*m*/*z* 476.1).

In addition, we found that IMM produced an obvious impurity after exposure to oxidative degradation, as shown in Figure 2(b), with the highest content reaching 10.42%. It was named impurity V. HPLC-UV and HPLC-ESI-MS analyses were performed to elucidate the structure and identify the mass of impurity V. The UV spectrum (*λ*_max_ 231.21 nm) of impurity V in HPLC-UV was similar to that of IMM (*λ*_max_ 229.90 nm), suggesting the presence of a diphenyl skeleton in impurity V. In the positive ion mode of HPLC-ESI-MS, impurity V and IMM showed their ion peaks at *m*/*z* 476.1 [M+H]^+^ and *m*/*z* 460.1 [M+H]^+^ (Figure 3), indicating a molecular weight difference of 16 between the two compounds. In combination with the difference in HPLC-ESI-MS data between impurity V and IMM, the structure of impurity V was assumed to be a nitrogen oxide by-product of IMM. To confirm this assumption, higher quantities of impurity V were needed. Thus, we extended the time of oxidative degradation from 24 h to 20 days. The reaction mixture was extracted with dichloromethane and water and then purified by silica gel column chromatography to yield impurity V with increased purity (HPLC analysis above 95%). The ^1^H NMR spectra data further revealed the structure of impurity V as 4-((7,7′-dimethoxy-5′-(methoxycarbonyl)-[4,4′-bibenzo[d][1,3]dioxol]-5-yl)methyl)morpholine 4-oxide, confirming our supposition that it was a by-product of excessive oxidation of IMM during synthesis.

### 3.2. Analytical Method Development

**Figure 4:**
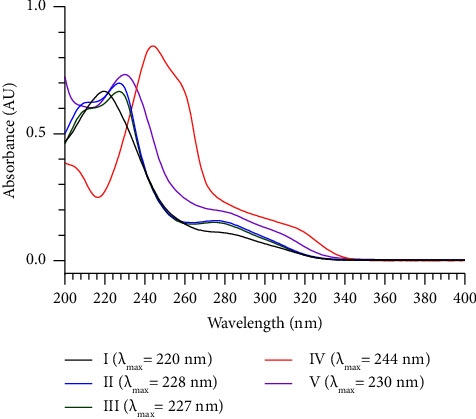
UV spectra of impurities I–V.

In this study, separating IMM and its impurities was critical given their structural and polarity similarities. In the preliminary work, C_8_ and C_18_ stationary phases were used for method development, and various mobile phases with different pH values, such as distinct proportions of acetonitrile-buffer and methanol-buffer solutions, were tested. Relatively, satisfactory results on symmetry and resolution were observed on the ODS C_18_ column by using ACN-0.1% (v/v) formic acid (pH adjusted to 4.0 with triethylamine). The isocratic elution mode was adopted at the beginning of the study; however, the separation between impurity I and IMM was only efficient with 50% acetonitrile, and the run time exceeded 80 min with 30% acetonitrile. Thus, the gradient elution mode was determined to be more suitable. The percentage of the organic phase was increased from 30% to 50% between 15 and 30 min to advance the Rt values of the impurities after the peak of IMM. In addition, the maximum absorption wavelengths of impurities I–V were 220, 228, 227, 244, and 230 nm (Figure 4), respectively. Thus, 230 nm was selected as the determination wavelength to improve the sensitivity of the method.

Among the five impurities, impurity V contained structurally alerted functional groups requiring additional scrutiny, and the estimated permitted level was set at 30 ppm. However, the above-mentioned HPLC method could not meet the requirements for the detection and quantification of impurity V at the 30-ppm level due to the low sensitivity of the UV detector. In this study, we utilized HPLC coupled with triple-quadrupole mass spectrometry to quantify this impurity.

Through multiple explorations of stationary and mobile phases, good peak separation was achieved on the Inertsil ODS-3 column (150 mm × 3.0 mm and particle size of 3 *μ*m) by using methanol, the mixture of 2.5 mmol/L ammonium formate and 0.05% formic acid solution at a constant proportion of 28 : 72 (v/v) as the mobile phase. The column temperature was controlled within 33°C–37°C, and the injection volume was 5 *μ*L. Specific quantitative ions 460.1 ⟶ 341 (*m*/*z*) for IMM and 476.1 ⟶ 373.2 (*m*/*z*) for impurity V were used for qualitative and quantitative analyses in accordance with the chemical structure and MS splitting decomposition law; the product ion spectra of [M+H]^+^ ions from IMM and impurity V obtained in the positive ion mode are shown in Supplementary Materials S12 and S13. The MS fragmentation pathways are shown in Table 1. The MS parameters were optimized using analytical software. Moreover, to protect the ESI source, the mobile phase and its eluents were transformed into waste at the time range of IMM elution.

To extract impurity V from IMM using the mixture of MeOH and water at a ratio of 30 : 70, ultrasonic and vibration treatments were applied; the latter was chosen because it is less harmful to the sample. We found inequalities in the amount of impurity V in each part of IMM and determined that IMM should be thoroughly ground for uniform mixing. We used repeatability assays to check whether extraction could be satisfactorily reproduced; the results are described in the next section.

### 3.3. Method Validation

The validation of the proposed methods for the quantification of impurities I–V was performed based on the criteria of ICH Q2, ICH Q3A, mirroring the referenced criteria in specificity, detection and quantification limits, linearity, accuracy, precision, and stability. The results from the validation experiments are summarized in Table 2.

#### 3.3.1. Specificity

**Figure 5:**
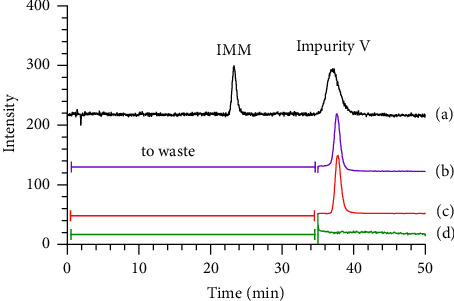
Typical MRM chromatograms showing (a) mixed solution of IMM and impurity V (3 ng/mL). Typical MRM chromatograms showing (b) IMM sample solution (0.1 mg/mL). (c) Standard solution of impurity V (3 ng/mL). (d) Blank.

The specificity of the methods was evaluated by injecting the blank and IMM with individual impurities. The corresponding chromatograms under optimal conditions are shown in Figures 2 and 5. The HPLC chromatograms indicated that the developed method could successfully separate each impurity (I–IV) from the other and from the main drug. The HPLC-MS/MS chromatograms demonstrated that the peak of IMM was so far from that of impurity V that was completely cut off from the waste and did not affect the detection of impurity V. Moreover, no interfering peaks were found in the blank solution, as revealed by the extracted ion chromatograms of impurity V in the precursor and fragment ions (*m*/*z* 476.1 ⟶ 373.2).

#### 3.3.2. System Suitability

The resolution between peaks of IMM and impurity I was used to assess system suitability of the HPLC method, since the chromatograms showed that the peak of impurity I was closest to the main peak, and the result was greater than 10. Similarly, the resolution between peaks of impurity V and IMM was greater than 10. The system suitability of both methods was adequate for the accurate analysis of impurities.

The evaluation of the specificity of the HPLC method revealed that impurity I was considerably increased under alkali degradation (1 M NaOH at 60°C), with a reduction of 7.14% of IMM at drug concentration. Meanwhile, impurity V increased sharply to 10.42% under strong oxidative degradation. The new drug IMM was relatively stable under other stress conditions, as listed in Table 3. In all forced conditions, the purity verification of all impurity peaks demonstrated the absence of interfering substances at the same retention time.

#### 3.3.3. Sensitivity

The LODs and LOQs of all impurities were estimated by injecting diluted solutions with known concentrations at S/N ratios of 3 : 1 and 10 : 1, respectively. The LODs and LOQs of impurities I–IV were 0.005 and 0.01 *μ*g/mL, respectively. For impurity V, the LOD and LOQ values were 0.05 and 0.17 ng/mL, respectively, which were equivalent to 0.05 and 0.17 ppm, respectively. These low LOQ values were adequate for the specific analysis and indicated that the methods with high sensitivity methods were satisfactory.

#### 3.3.4. Linearity

Linearity was evaluated at seven concentration levels for each impurity. The linear range was 0.10–2.03 *μ*g/mL for impurities I–IV, and the linear range for impurity V was 0.34–27.31 ng/mL due to its high detection limit concentration requirement. The slope, intercept, and regression coefficients were calculated using the least-squares linear regression analysis, as shown in Table 2. The methods showed linearity across the calibration range for all impurities, and the correlation coefficients were 0.9999, 0.9999, 0.9998, 0.9999, and 0.9999 for impurities I–V, respectively.

#### 3.3.5. Accuracy

Accuracy was evaluated through spiked recovery experiments. Authentic impurities were spiked into sample solutions in triplicate by using concentration levels of 50%, 100%, and 150%. Three determinations were performed for each level. The data for recovery were calculated by comparing the experimental and theoretical values. Recoveries in the range of 91.18%–111.27% with RSD values below 3.69% were achieved for all impurities, and the detailed data are shown in Table 2.

#### 3.3.6. Precision and Stability

The precision and repeatability results were expressed as RSD% and are presented in Table 2. The variability of the results was low, with RSD% values being less than 1.08% for precision and 5.22% for repeatability in the quantitative determination of impurities I–IV. For impurity V, the RSD% values of precision and repeatability were 0.36% and 8.72%, respectively, indicating high precision of the developed methods. The stability of the standard and sample solutions was established through analysis at different time intervals within 24 h. The RSD values of all impurities were less than 4.04% within 24 h. The results summarized in Table 2 demonstrate reliability of our methods for impurity quantification.

### 3.4. Toxicity Prediction Studies of Impurity V

The potential tumorigenic effect of impurity V was further evaluated using ADMET 2.0, preADMET, and pkCSM computer programs available online and through Toxtree software. The prediction results showed potential carcinogenicity (nongenotoxicity) in impurity V by using ADMET 2.0, preADMET, and Toxtree software. However, the pkCSM program predicted no carcinogenicity. Although impurity V with structurally alerting functional groups (N-oxide) has no risk of mutagenicity as predicted by software, there is still a potential risk of genetic mutations, or rearrangements in mammalian cell systems NDA changes, considering that IMM requires long-term administration and the nitrogen oxide moiety is highly electrophilic [33, 34]. To ensure the safety of the new drug for clinical use, impurity V was still controlled below the “TTC” level.

### 3.5. Sample Analysis

The validated methods were applied to measure the impurities in three batches of IMM API samples. The contents of impurities I–IV were quantified with the external standard method by calculating the peak areas; the results are listed in Table 4. All impurities were below the defined acceptable limits, indicating that all of them were well controlled under established conditions.

### 3.6. Greenness Evaluation of the Proposed Methods

**Figure 6:**
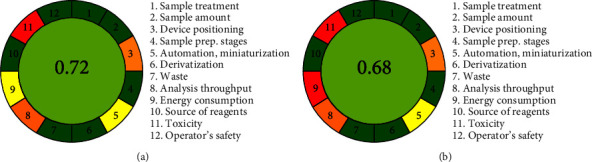
Greenness assessment of the proposed methods according to AGREE tools: (a) HPLC method and (b) HPLC-MS/MS method.

The greenness assessment of the proposed methods was evaluated by using AGREE software, and the AGREE pictogram score values of 0.72 and 0.68 are shown in Figure 6. The HPLC-MS/MS method consumes more energy than the HPLC method, because it requires HPLC-MS grade solvent and N_2_ with high purity in large amounts. The results showed that both methods were ecofriendly and economical with high scores, although methanol, acetonitrile, HCl, NaOH, and H_2_O_2_ have some toxicity and should be treated regularly in order to prevent any potentially unsafe exposure to the environment.

## 4. Conclusion

Five trace impurities in IMM, an innovative bulk drug for the treatment of NAFLD, were identified and analyzed by applying the HPLC-MS/MS technique together with spectral and classical synthetic methods. Among these impurities, impurity V belonged to nitrogen oxides with strong electrophilicity, and the toxicity prediction study, using various pieces of software, suggested a potential carcinogenicity with this impurity. In accordance with ICH guidelines, a reliable HPLC method and a sensitive HPLC-MS/MS method were developed and optimized for the quantitative analysis of the five trace impurities in IMM APIs. In terms of methodology, the methods proved to be specific, precise, accurate, and linear within the assessed concentration range. Validation and greenness assessment proved that the proposed methods are specific, accurate, sensitive, ecofriendly, and economical green analysis. Hence, they can be applied to in-process monitoring of impurities during pharmaceutical manufacturing. Quantitative determination of the impurities in several batches of IMM APIs revealed the high efficiency of these methods even at a low concentration level, validating this methodology in the evaluation of drug safety during clinical treatment. Our systematic study involving the identification and determination of impurities provides a basis for further scientific research on IMM APIs and lays a foundation for the continued advancement of impurity detection methods applied to novel drugs with a similar structure discovery process. This study contributes to the quantitative evaluation of IMM active pharmaceutical ingredients and provides a scientific basis for the effective development of innovative drugs for the treatment of NAFLD.

## Figures and Tables

**Table 1:** MS fragment pathways and quantitative ions of IMM and impurity V.

No	Code	MRM (*m*/*z*)	MS fragment pathways
1	IMM	460.1 ⟶ 341	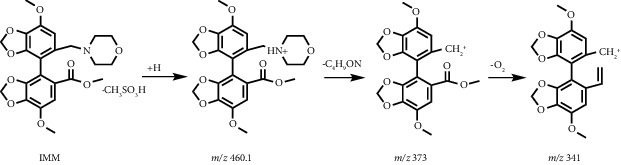

2	Impurity V	476.1 ⟶ 373.2	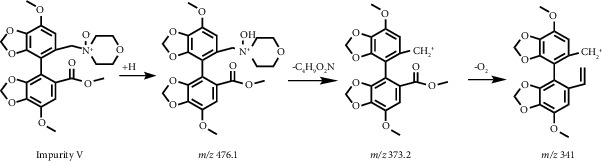

**Table 2:** Summary of the validation report of the proposed methods.

Parameters	I	II	III	IV	V
Linear equation	*y* = 1.7418*x* − 0.0134	*y* = 2.6599*x* − 0.0328	*y* = 2.5719*x* − 0.0266	*y* = 1.7827*x* − 0.0168	*y* = 165113*x* − 26088
*R*	0.9999	0.9999	0.9998	0.9999	0.9999
Linearity range (*μ*g/mL)	0.10–2.03	0.10–2.03	0.10–2.03	0.10–2.03	0.34–27.31 ng/mL
LOD (*μ*g/mL)	0.005	0.005	0.005	0.005	0.05 ng/mL
LOQ (*μ*g/mL)	0.01	0.01	0.01	0.01	0.17 ng/mL
Precision (%) (*n* = 6)	0.75	1.08	0.42	1.05	0.36
Repeatability (%) (*n* = 6)	3.65	3.36	2.25	5.22	8.72
Stability (%)	2.10	4.04	1.53	2.55	0.57
*Accuracy at low level* (*n* = 3)					
Recovery (%)	102.99	101.96	104.80	100.98	91.18
RSD (%)	3.09	3.09	3.64	2.30	1.89
*Accuracy at medium level* (*n* = 3)					
Recovery (%)	102.68	98.35	104.20	101.69	111.27
RSD (%)	3.69	0.70	1.83	1.78	0.58
*Accuracy at high level* (*n* = 3)					
Recovery (%)	102.53	100.84	105.78	103.28	102.96
RSD (%)	2.28	1.89	1.93	1.83	0.73

**Table 3:** Percentage of degradation of IMM under different stress conditions.

Impurities	*Stress conditions* (%)					
	Undegraded sample	Acid (1 M at 60°C)	Basic (1 M at 60°C)	Hydrogen peroxide (30% at 60°C)	Thermal (60°C)	Photodegradation (4500 Lx ± 500 Lx)
I	0.01	0.31	7.14	0.05	0.01	0.09
II	0.01	0.04	N.D.	N.D.	0.01	0.01
III	0.07	0.02	0.07	0.08	0.08	0.08
IV	N.D.	N.D.	N.D.	N.D.	N.D.	N.D.
V	N.D.	N.D.	N.D.	10.42	N.D.	N.D.
Unknown	0.01	0.08	0.01	N.D.	0.06	0.20
IMM	99.89	99.54	92.78	89.45	99.85	99.62

N.D.: not detected.

**Table 4:** Amount of impurities in IMM API samples.

Batch no	*Impurities* (%)				
	I	II	III	IV	V
20211001	0.0065	0.0116	0.0089	N.D.	0.0020
20211101	0.0078	0.0100	0.0072	N.D.	0.0022
20220622	0.0160	N.D.	0.0376	N.D.	0.0024

N.D.: not detected.

## Data Availability

The data used to support the findings of this study are included within the article and the supplementary information file.

## Supplementary Materials

Supplementary Materials20230730-version The extra supporting data are collected in Supplementary Materials file that included the spectrum data of IMM and impurities, product ion spectra of [M+H]^+^ ions from IMM and impurity V, HPLC chromatograms of I–IV, and MRM chromatograms of V for API samples.
