# Supplementary material for: Identification and Determination of Impurities in a New Therapeutic Agent for Fatty Liver Disease
Source: J Anal Methods Chem. 2023 Aug 4;2023:3116223. doi: 10.1155/2023/3116223 (PMC10421711; doi:10.1155/2023/3116223)
Supplement: Supplementary Materials20230730-version — The extra supporting data are collected in Supplementary Materials file that included the spectrum data of IMM and impurities, product ion spectra of [M+H]+ ions from IMM and impurity V, HPLC chromatograms of I–IV, and MRM chromatograms of V for API samples. [file 3116223.f1.docx]

**Supplementary Materials**

**Identification and Determination of Impurities in a New Therapeutic Agent for Fatty Liver Disease**

Huihui Shao, ^1†^ Jing Feng,^1†^ Hanyilan Zhang,^1^ Yuanyuan Zhang,^1^ Tong Qin,^1^ Yuhua Hu,^1^ Wenxuan Zhang,^1^ Tiesong Wang,^2^ Song Wu,^1^ Qingyun Yang^1^

*^1^ State Key Laboratory of Bioactive Substance and Function of Natural Medicines, Institute of Materia Medica, Chinese Academy of Medical Sciences & Peking Union Medical College, Beijing 100050, China*

*^2^ NMPA Key Laboratory for Research and Evaluation of Generic Drugs, Beijing Institute for Drug Control, Beijing 102206, China*

Correspondence should be addressed to Qingyun Yang; yqy@imm.ac.cn and Song Wu; ws@imm.ac.cn

Telephone: +86-010-8316-3542, Fax: +86-010-6301-7757

**Contents**

[S1. ^1^H NMR, ^13^C NMR, and HR-ESI–MS spectral data of IMM 1](#_Toc137321930)

[S2. ^1^H NMR, ^13^C NMR, and HR-ESI–MS spectral data of impurity I 3](#_Toc137321931)

[S3. Chromatogram of purity analysis for impurity I (96.78%) 4](#_Toc137321932)

[S4. ^1^H NMR, ^13^C NMR, and HR-ESI–MS spectral data of impurity II 5](#_Toc137321933)

[S5. Chromatogram of purity analysis for impurity II (99.64%) 6](#_Toc137321934)

[S6. ^1^H NMR, ^13^C NMR, and HR-ESI–MS spectral data of impurity III 7](#_Toc137321935)

[S7. Chromatogram of purity analysis for impurity III (99.18%) 8](#_Toc137321936)

[S8. ^1^H NMR, ^13^C NMR, and HR-ESI–MS spectral data of impurity IV 9](#_Toc137321937)

[S9. Chromatogram of purity analysis for impurity IV (99.66%) 10](#_Toc137321938)

[S10. ^1^H NMR, ^13^C NMR, and HR-ESI–MS spectral data of impurity V 11](#_Toc137321939)

[S11. Chromatogram of purity analysis for impurity V (95.63%) 12](#_Toc137321940)

[S12. Product ion spectra of [M+H]^+^ ions from impurity V 13](#_Toc137321941)

[S13. Product ion spectra of [M+H]^+^ ions from IMM 13](#_Toc137321942)

[S14. HPLC chromatograms of impurities Ⅰ–IV (Batch nos: 20211001, 20211101, 20220622) 14](#_Toc137321943)

[S15. MRM chromatograms of impurity Ⅴ (Batch nos: 20211001, 20211101, 20220622) 14](#_Toc137321944)

# S1. ^1^H NMR, ^13^C NMR, and HR-ESI–MS spectral data of IMM

Methyl 7, 7'-dimethoxy-5'-(morpholinomethyl)-[4, 4'-bibenzo[d][1,3]dioxole]-5-carboxylate

methanesulfonate

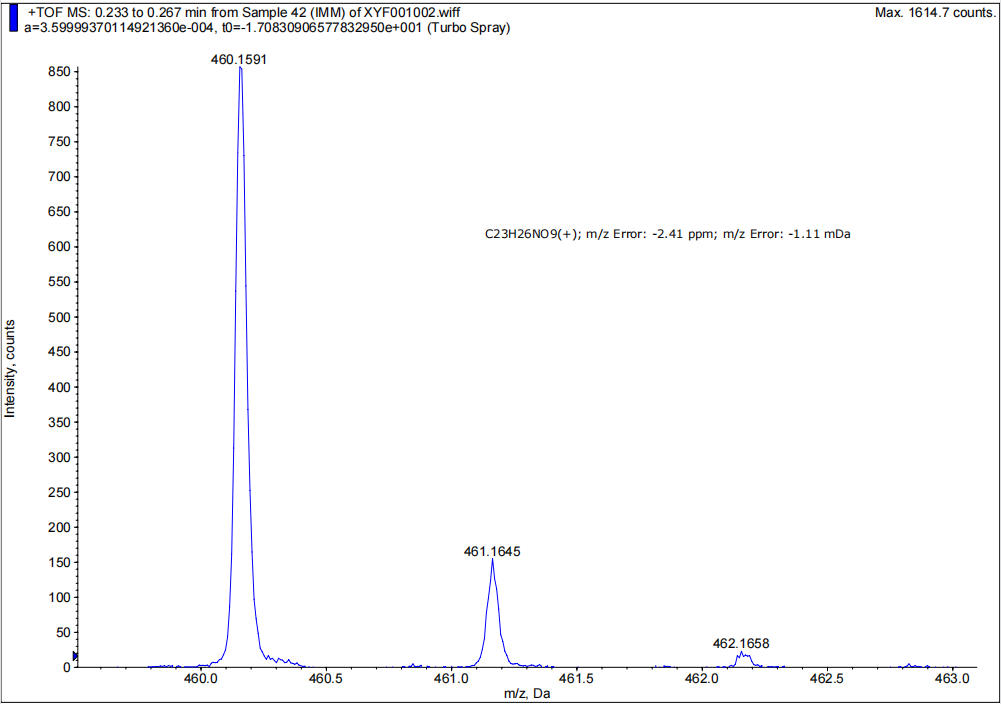


^1^H NMR (400 MHz, CDCl_3_) *δ* 11.08 (s, 1H), 7.36 (s, 1H), 7.33 (s, 1H), 6.08 (d, *J* = 1.2 Hz, 1H), 5.96 (d, *J* = 1.2 Hz, 1H), 5.91 (d, *J* = 1.4 Hz, 1H), 5.89 (d, *J* = 1.4 Hz, 1H), 4.33 (dd, *J* = 13.2, 4.2 Hz, 1H), 4.08 (m, *J* = 13.7, 11.8, 2.2 Hz, 1H), 4.02 (s, 3H), 3.98 (s, 3H), 3.92 (m, *J* = 12.3, 11.5, 1.8 Hz, 1H), 3.87 (d, *J* = 6.6 Hz, 1H), 3.85 – 3.82 (m, 1H), 3.79 (dd, *J* = 13.1, 3.4 Hz, 1H), 3.71 (s, 3H), 3.55 (d, *J* = 12.1 Hz, 1H), 3.35 (d, *J* = 12.1 Hz, 1H), 2.82 (s, 3H), 2.67 (m, *J* = 12.1, 8.8, 3.8 Hz, 1H), 2.48 – 2.36 (m, 1H). ^13^C NMR (101 MHz, CDCl_3_) *δ* 166.26, 147.29, 146.87, 144.39, 143.30, 138.48, 135.94, 124.55, 120.67, 112.05, 111.58, 110.93, 109.37, 102.64, 102.12, 63.78, 63.75, 57.94, 57.10, 56.85, 52.46, 52.40, 50.73, 39.54. HR-ESI–MS: *m/z* [M–CH_3_SO_3_H+H]^+^ (organic base) calcd for C_23_H_25_NO_9_ 460.1529, found 460.1591.

# S2. ^1^H NMR, ^13^C NMR, and HR-ESI–MS spectral data of impurity I

7,7'-dimethoxy-5'-(morpholinomethyl)-[4,4'-bibenzo[d][1,3]dioxole]-5-carboxylic acid

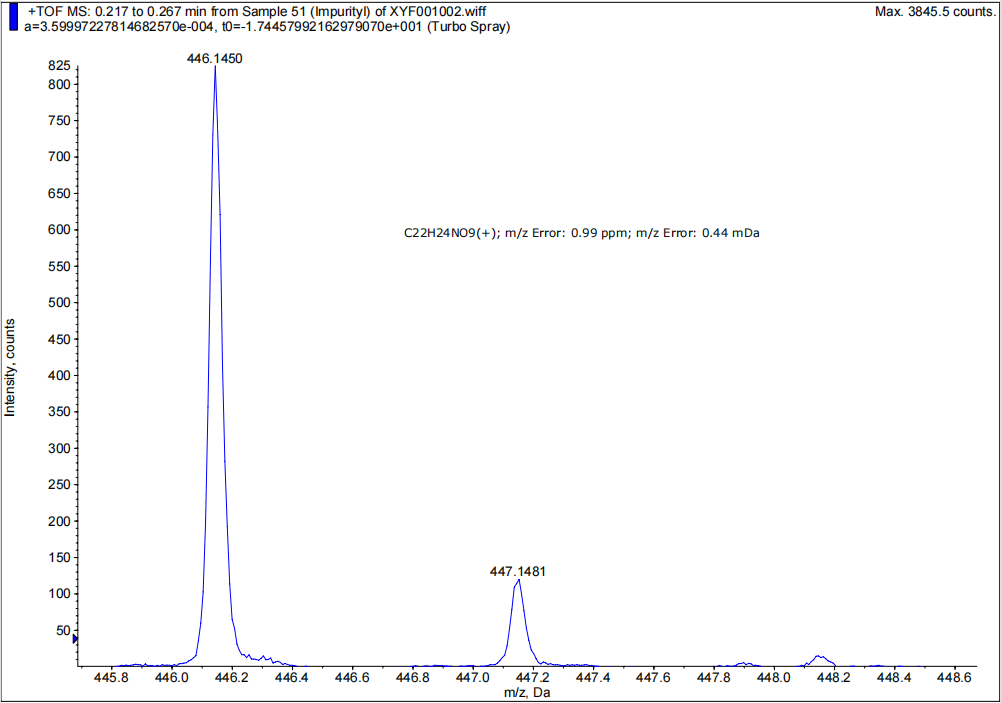


^1^H NMR (400 MHz, CDCl_3_) *δ* 10.41 (s, 1H), 7.66 (s, 1H), 7.54 (s, 1H), 6.01 (dd, *J* = 20.2, 1.2 Hz, 2H), 5.86 (dd, *J* = 16.1, 1.3 Hz, 2H), 4.11 – 4.03 (m, 1H), 4.01 (s, 3H), 3.98 (s, 3H), 3.96 – 3.69 (m, 4H), 3.65 – 3.41 (m, 2H), 3.32 (d, *J* = 12.5 Hz, 1H), 2.46 (d, *J* = 40.9 Hz, 2H). ^13^C NMR (101 MHz, DMSO-*d*_6_) *δ* 167.40, 147.67, 146.90, 143.10, 142.66, 138.43, 135.16, 125.46, 122.32, 112.67, 111.85, 109.33, 102.94, 101.98, 63.30, 57.03, 56.89, 56.78. HR-ESI–MS: *m/z* [M+H]^+^ calcd for C_22_H_23_NO_9_ 446.1373, found 446.1450.

# S3. Chromatogram of purity analysis for impurity I (96.78%)


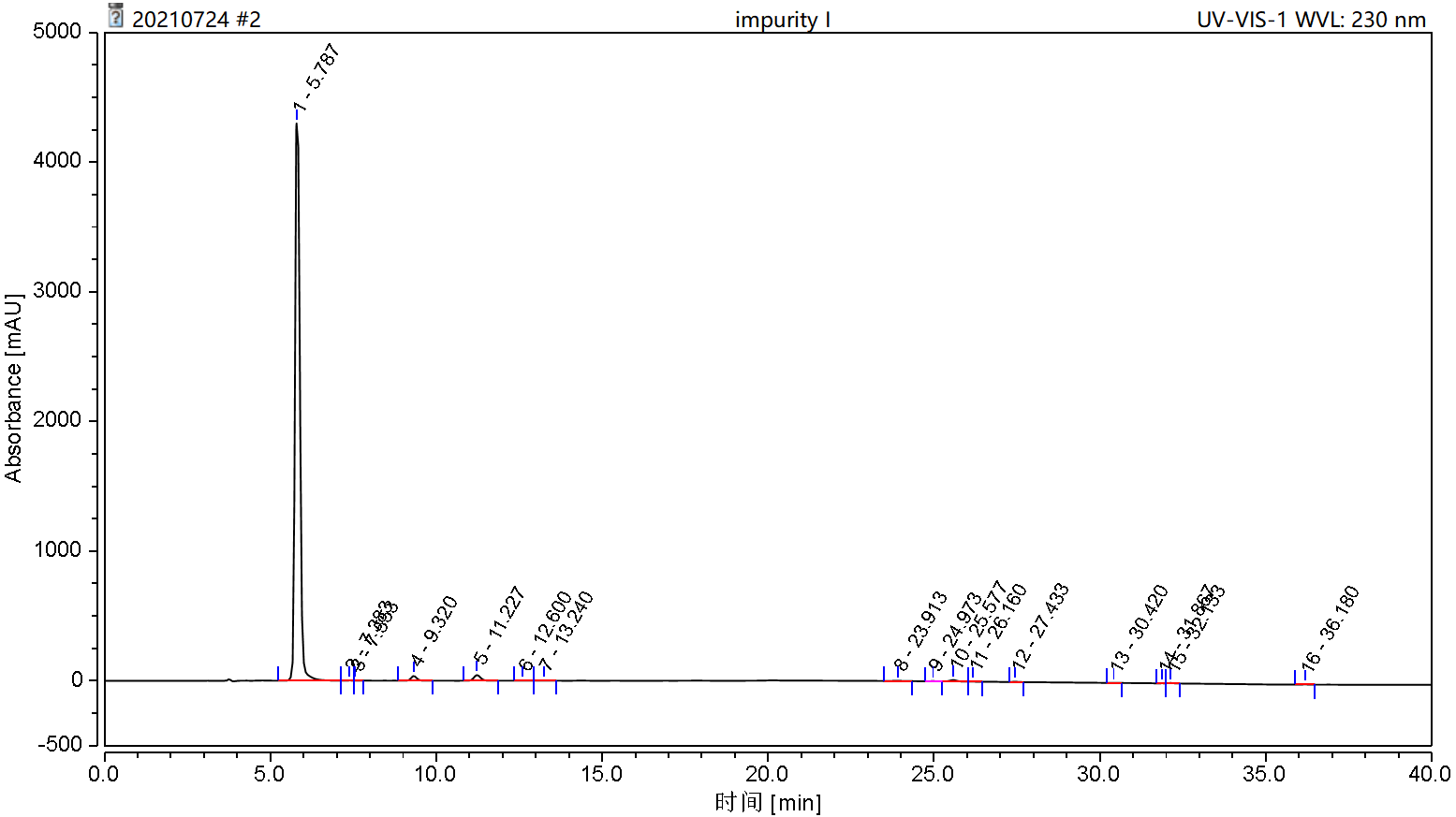


# S4. ^1^H NMR, ^13^C NMR, and HR-ESI–MS spectral data of impurity II

Methyl 5'-(hydroxymethyl)-7,7'-dimethoxy-[4,4'-bibenzo[d][1,3]dioxole]-5-carboxylate

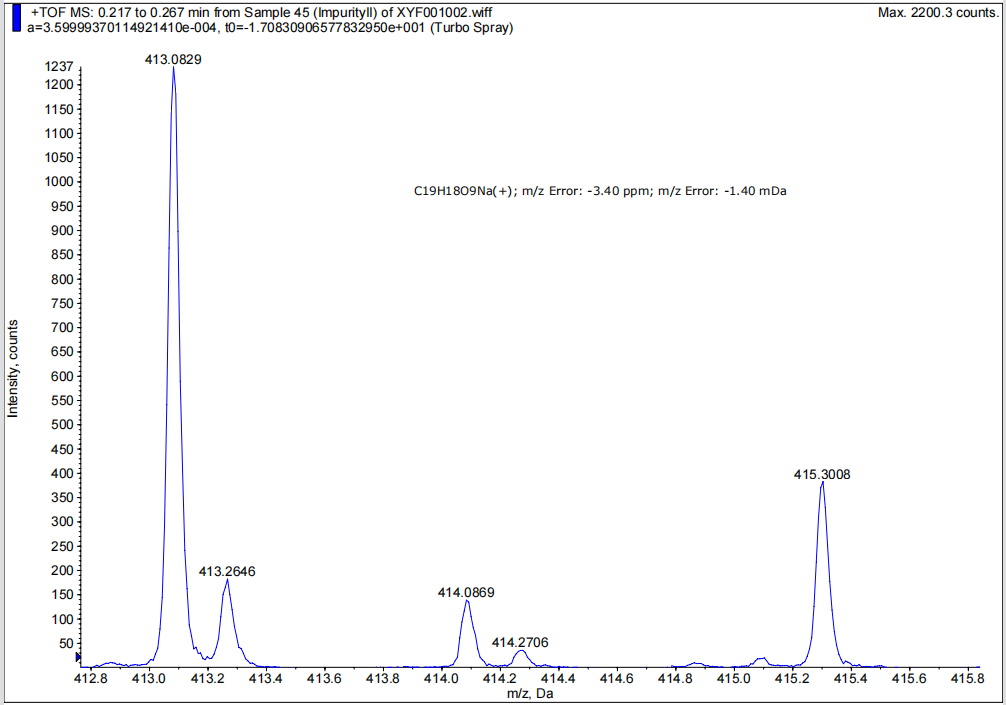


^1^H NMR (400 MHz, CDCl_3_) *δ* 7.33 (s, 1H), 6.77 (s, 1H), 6.03 (q, 2H), 5.91 (s, 2H), 4.37 (q, 2H), 3.97 (s, 3H), 3.94 (s, 3H), 3.71 (s, 3H). ^13^C NMR (101 MHz, CDCl_3_) *δ* 166.97, 147.64, 146.52, 143.46, 142.86, 138.35, 134.31, 133.70, 124.57, 111.46, 110.65, 109.03, 108.10, 102.42, 101.74, 63.56, 56.71, 56.41, 52.31. HR-ESI–MS: *m/z* [M+Na]^+^ calcd for C_19_H_18_O_9_ 413.0951, found 413.0829.

# S5. Chromatogram of purity analysis for impurity II (99.64%)


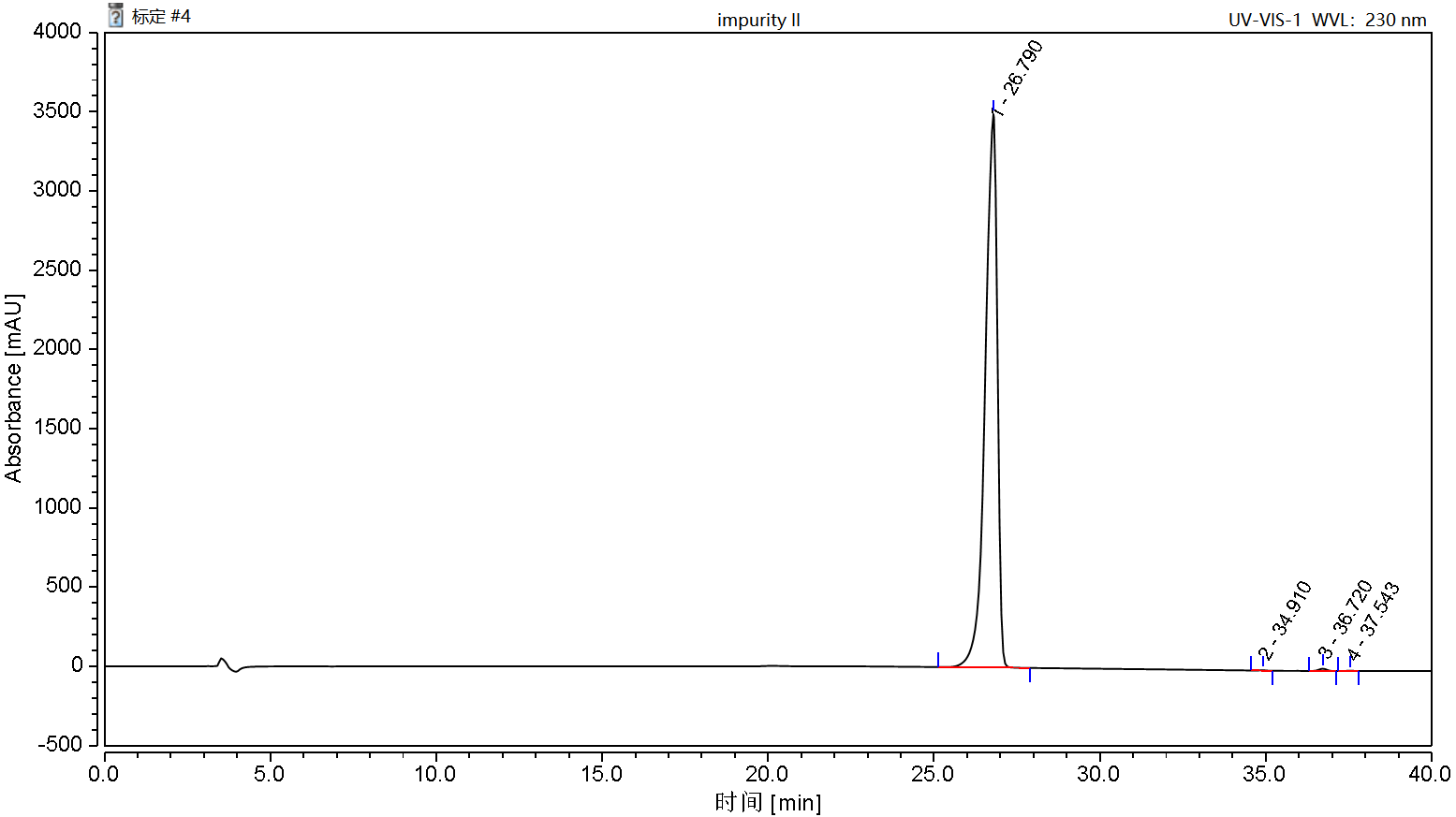


# S6. ^1^H NMR, ^13^C NMR, and HR-ESI–MS spectral data of impurity III

Methyl 7,7'-dimethoxy-5'-(methoxymethyl)-[4,4'-bibenzo[d][1,3]dioxole]-5-carboxylate

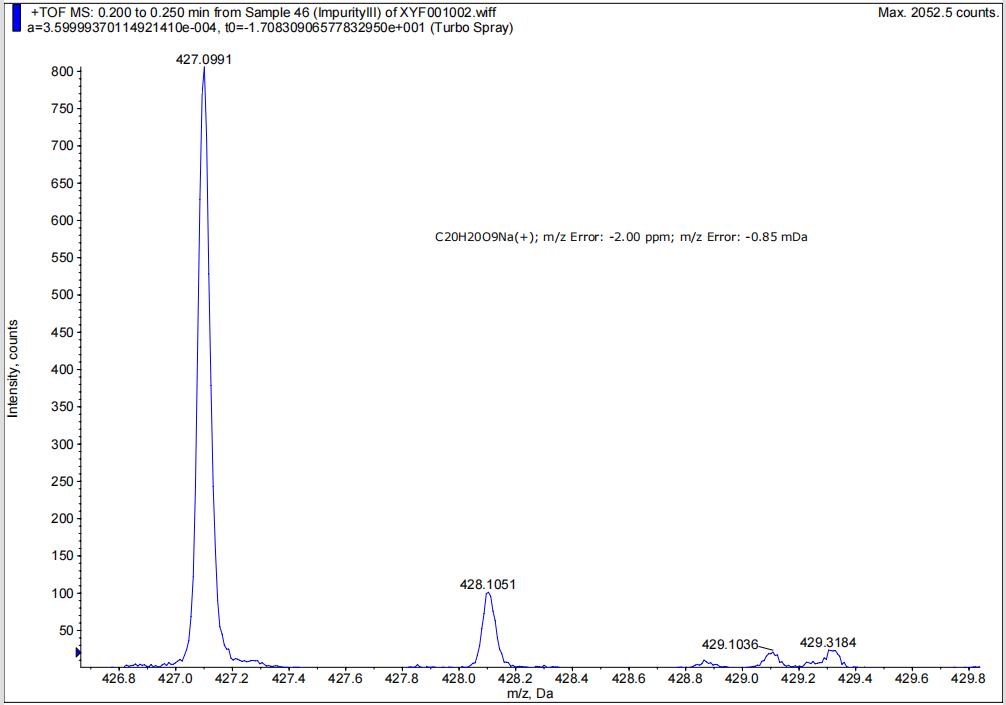


^1^H NMR (400 MHz, CDCl_3_) *δ* 7.36 (s, 1H), 6.72 (s, 1H), 6.02 (s, 2H), 5.92 (s, 2H), 4.16 (q, 2H), 3.97 (s, 3H), 3.94 (s, 3H), 3.65 (s, 3H), 3.20 (s, 3H). ^13^C NMR (101 MHz, CDCl_3_) *δ* 166.48, 147.61, 146.57, 143.16, 142.73, 138.15, 134.00, 130.98, 124.68, 111.25, 110.67, 109.58, 107.21, 102.40, 101.66, 72.11, 57.81, 56.64, 56.42, 52.01. HR-ESI–MS: *m/z* [M+Na]^+^ calcd for C_20_H_20_O_9_ 427.1107, found 427.0991.

# S7. Chromatogram of purity analysis for impurity III (99.18%)


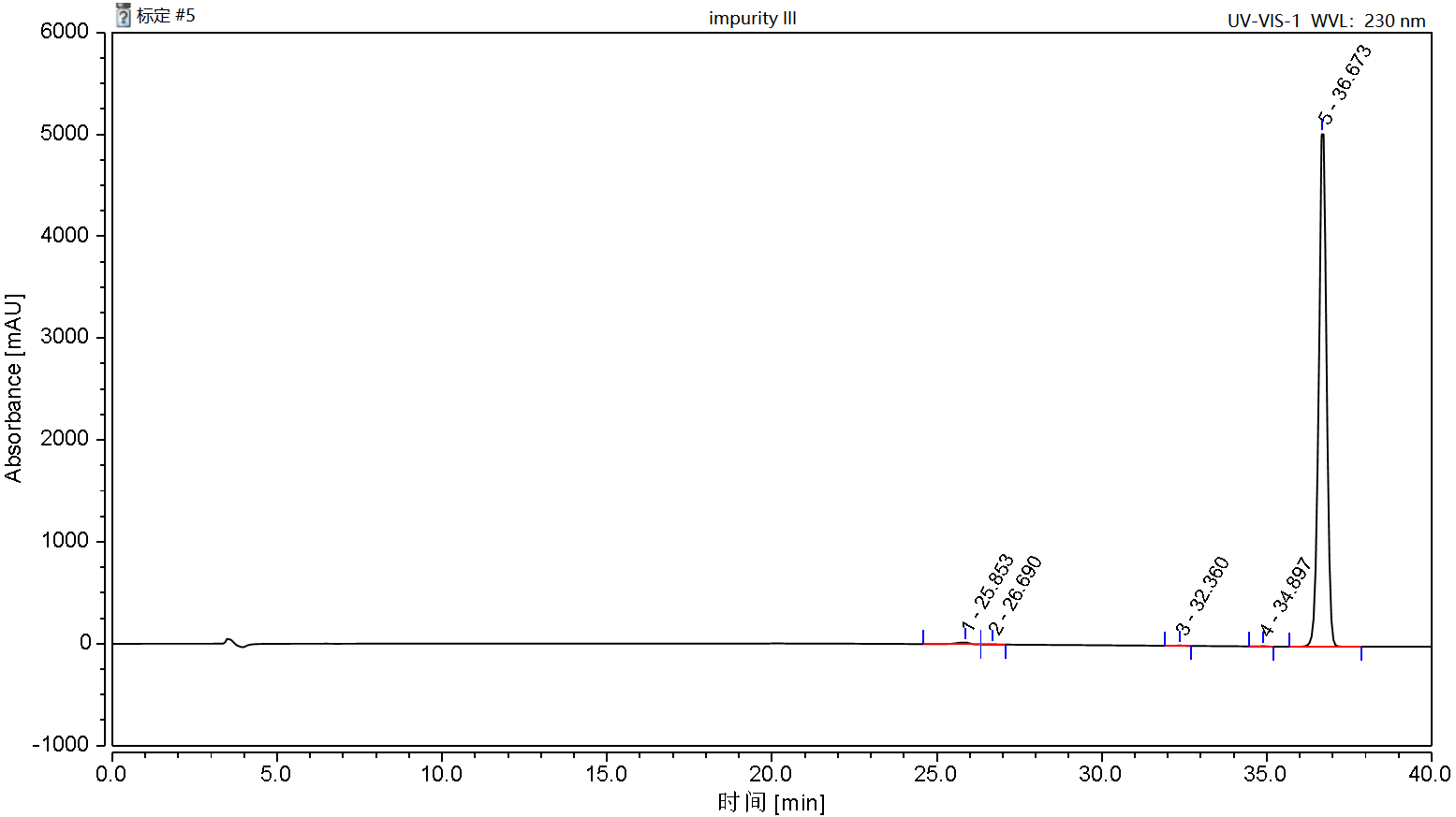


# S8. ^1^H NMR, ^13^C NMR, and HR-ESI–MS spectral data of impurity IV

4,10-dimethoxy-[1,3]dioxolo[4',5':3,4]benzo[1,2-*c*][1,3]dioxolo[4',5':5,6]benzo[1,2-*e*]oxepin-6(8H)-one

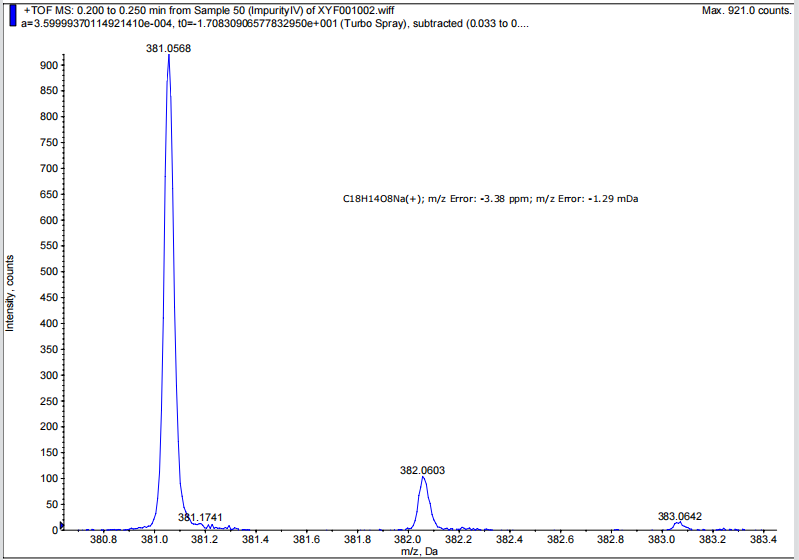


^1^H NMR (400 MHz, CDCl_3_) *δ* 7.21 (s, 1H), 6.67 (s, 1H), 6.17 (d, *J* = 1.4 Hz, 1H), 6.11 (d, *J* = 1.5 Hz, 1H), 6.08 (d, *J* = 1.5 Hz, 1H), 5.99 (d, *J* = 1.4 Hz, 1H), 5.03 (d, *J* = 12.2 Hz, 1H), 4.83 (d, *J* = 12.2 Hz, 1H), 3.97 (s, 3H), 3.95 (s, 3H). ^13^C NMR (101 MHz, CDCl_3_) *δ* 169.57, 146.95, 145.97, 143.41, 138.51, 136.40, 129.67, 124.65, 111.67, 109.12, 108.93, 108.21, 102.53, 102.11, 69.43, 56.87, 56.69. HR-ESI/APCI–MS: *m/z* [M+H]^+^ calcd for C_18_H_14_O_8_ 381.0689, found 381.0568.

# S9. Chromatogram of purity analysis for impurity IV (99.66%)


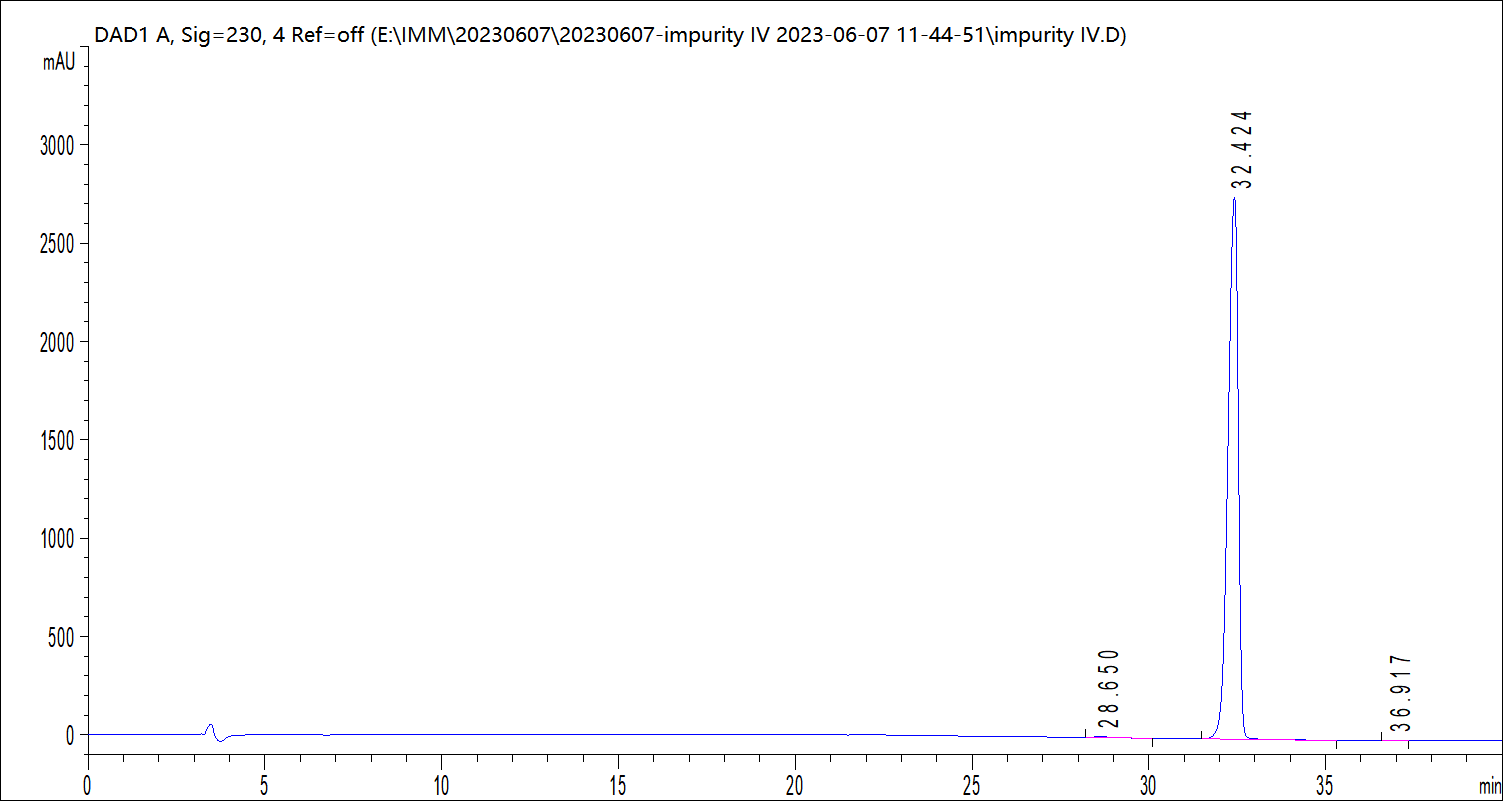


# S10. ^1^H NMR, ^13^C NMR, and HR-ESI–MS spectral data of impurity V

4-((7,7'-dimethoxy-5'-(methoxycarbonyl)-[4,4'-bibenzo[*d*][1,3]dioxol]-5-yl)methyl)morpholine 4-oxide

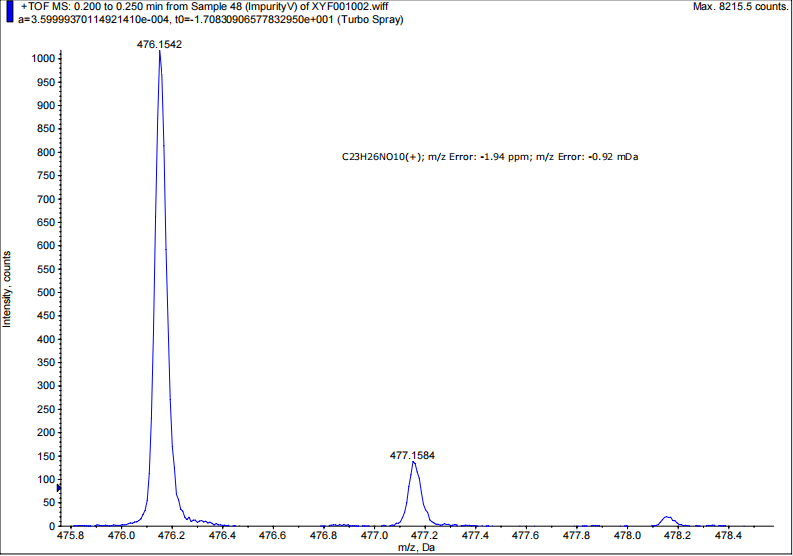


^1^H NMR (600 MHz, DMSO-*d*_6_) *δ* 7.30 (d, *J* = 4.4 Hz, 2H), 6.09 (d, *J* = 7.2 Hz, 2H), 5.94 (s, 1H), 5.87 (s, 1H), 4.24 (d, *J* = 12.9 Hz, 1H), 4.11 – 4.01 (m, 2H), 3.98 (t, *J* = 11.6 Hz, 1H), 3.91 (s, 3H), 3.85 (s, 3H), 3.62 (s, 3H), 3.58 – 3.40 (m, 4H), 2.67 (d, *J* = 11.8 Hz, 1H), 2.60 (d, *J* = 11.3 Hz, 1H). ^13^C NMR (151 MHz, DMSO-*d*_6_) *δ* 166.25, 147.49, 146.58, 142.65, 142.32, 138.69, 135.20, 124.33, 114.29, 122.72, 112.52, 111.65, 110.17, 102.94, 101.85, 71.94, 63.54, 61.74, 61.31, 61.25, 56.81, 56.66, 52.38. HR-ESI–MS: *m/z* [M+H]^+^ calcd for C_23_H_25_NO_10_ 476.1478 found 476.1542.

# S11. Chromatogram of purity analysis for impurity V (95.63%)


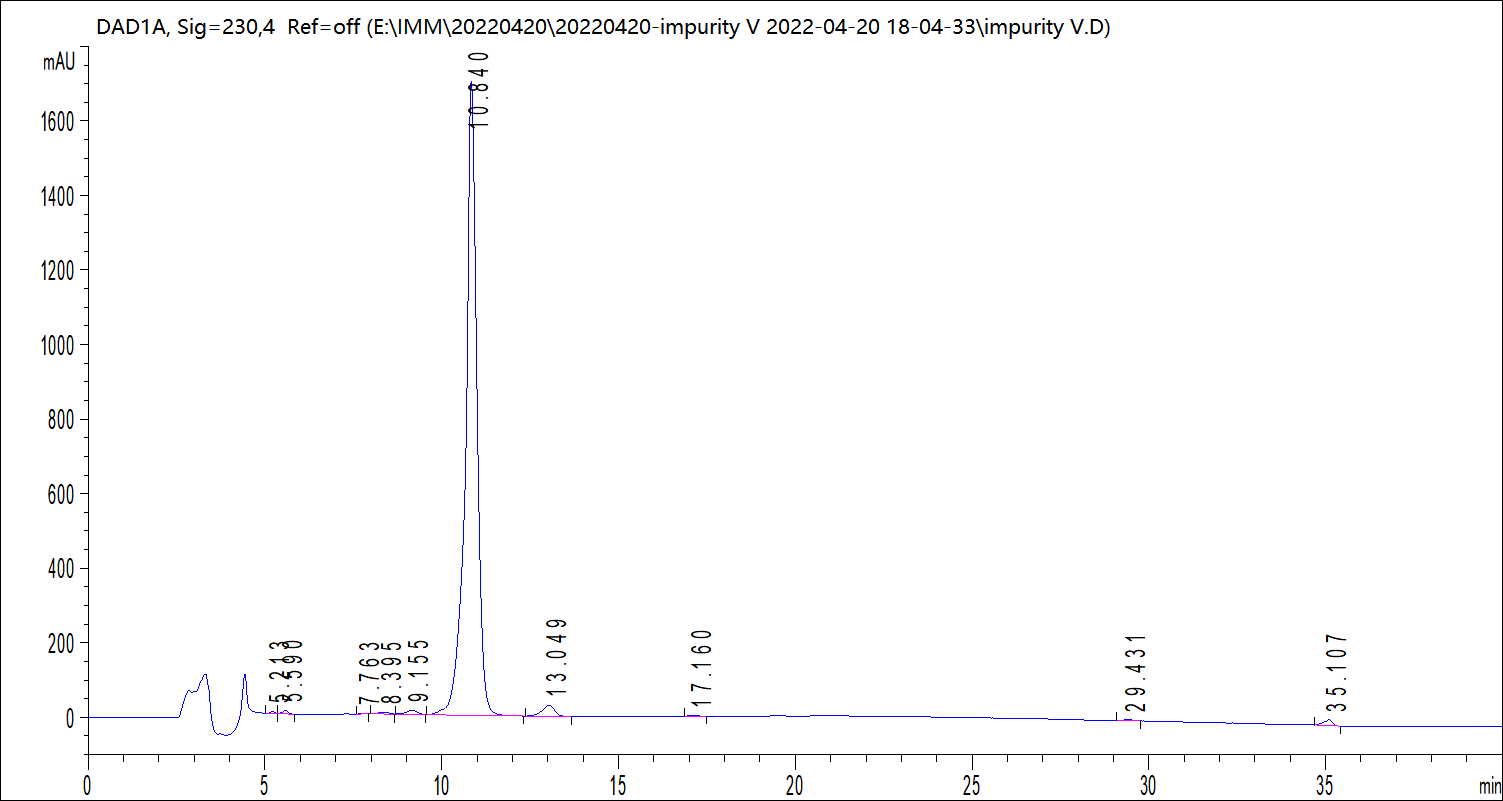


# S12. Product ion spectra of [M+H]^+^ ions from impurity V

# S13. Product ion spectra of [M+H]^+^ ions from IMM

# S14. HPLC chromatograms of impurities Ⅰ–IV (Batch nos: 20211001, 20211101, 20220622)

# S15. MRM chromatograms of impurity Ⅴ (Batch nos: 20211001, 20211101, 20220622)
